# 2-Amino-4-(4-chloro­phen­yl)-5,6,7,8,9,10-hexa­hydro­benzo[8]annulene-1,3-dicarbonitrile

**DOI:** 10.1107/S1600536812034861

**Published:** 2012-08-11

**Authors:** V. Rajni Swamy, N. Srinivasan, R. Ranjith Kumar, R.V. Krishnakumar

**Affiliations:** a Department of Physics, Thiagarajar College, Madurai 625 009, India; b School of Chemistry, Madurai Kamaraj University, Madurai 625 021, India

## Abstract

In the title compound, C_20_H_18_ClN_3_, the cyclo­octene ring exhibits conformational disorder of two methyl­ene groups with a site-occupation factor of 0.859 (6) for the major occupied site. In the crystal, mol­ecules are connected into inversion dimers *via* pairs of weak N—H⋯N hydrogen bonds, forming an *R*_2_^2^(12) graph-set motif. These dimers are further connected *via* weak N—H⋯Cl inter­actions into chains running along [011]. There are also C—H⋯N interactions present in the crystal.

## Related literature

For hydrogen-bond motifs, see: Bernstein *et al.* (1995 ▶). For puckering parameters, see: Cremer & Pople (1975 ▶) For conformational analysis of rings, see: Allen *et al.* (1996 ▶); Evans & Boeyens (1988 ▶, 1989 ▶); Hendrickson (1967 ▶).
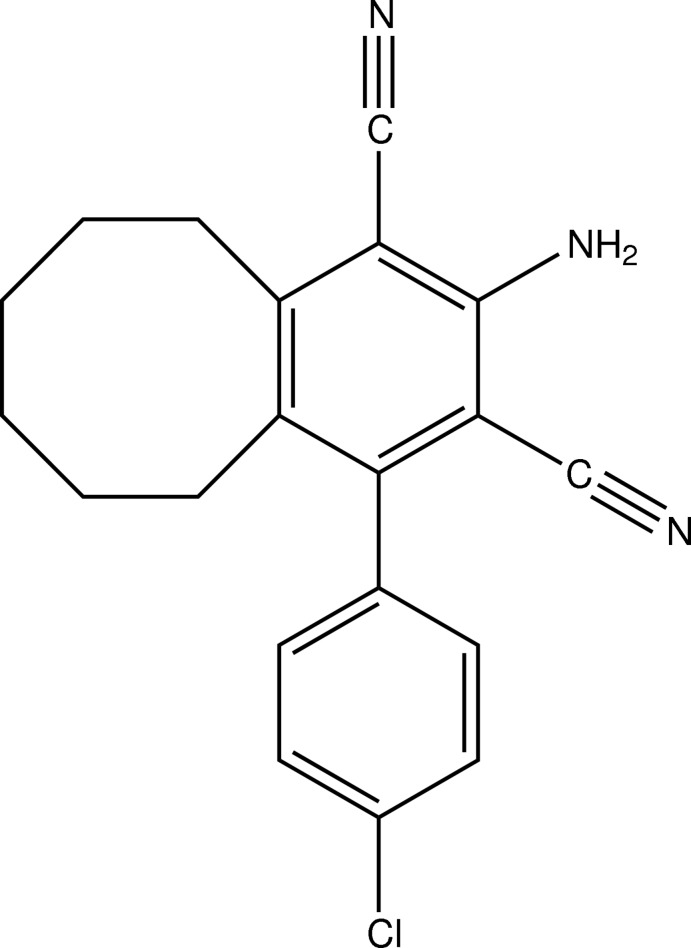


## Experimental

### 

#### Crystal data

C_20_H_18_ClN_3_
*M* *_r_* = 335.82
Orthorhombic, 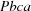

*a* = 11.3835 (9) Å
*b* = 16.9840 (13) Å
*c* = 18.4766 (14) Å
*V* = 3572.2 (5) Å^3^
*Z* = 8
Mo *K*α radiation
μ = 0.22 mm^−1^
*T* = 298 K
0.28 × 0.13 × 0.10 mm

#### Data collection

Bruker Kappa APEXII CCD diffractometer
Absorption correction: multi-scan (*SADABS*; Bruker, 2009 ▶) *T*_min_ = 0.966, *T*_max_ = 0.978
39132 measured reflections
4279 independent reflections
3440 reflections with *I* > 2σ(*I*)
*R*_int_ = 0.025

#### Refinement

*R*[*F* ^2^ > 2σ(*F* ^2^)] = 0.045
*wR*(*F* ^2^) = 0.137
*S* = 1.04
4279 reflections
226 parameters
H-atom parameters constrained
Δρ_max_ = 0.25 e Å^−3^
Δρ_min_ = −0.23 e Å^−3^

### 

Data collection: *APEX2* (Bruker, 2009 ▶); cell refinement: *SAINT* (Bruker, 2009 ▶); data reduction: *SAINT*; program(s) used to solve structure: *SHELXS97* (Sheldrick, 2008 ▶); program(s) used to refine structure: *SHELXL97* (Sheldrick, 2008 ▶); molecular graphics: *PLUTON* (Spek, 2009 ▶); software used to prepare material for publication: *SHELXL97*.

## Supplementary Material

Crystal structure: contains datablock(s) I, global. DOI: 10.1107/S1600536812034861/bt5985sup1.cif

Structure factors: contains datablock(s) I. DOI: 10.1107/S1600536812034861/bt5985Isup2.hkl

Supplementary material file. DOI: 10.1107/S1600536812034861/bt5985Isup3.cml

Additional supplementary materials:  crystallographic information; 3D view; checkCIF report

## Figures and Tables

**Table 1:** Hydrogen-bond geometry (Å, °)

*D*—H⋯*A*	*D*—H	H⋯*A*	*D*⋯*A*	*D*—H⋯*A*
N1—H1*B*⋯N3^i^	0.86	2.44	3.1636 (18)	142
C11′—H11*C*⋯N2^ii^	0.97	2.57	3.319 (16)	135
N1—H1*A*⋯Cl1^iii^	0.86	2.87	3.6628 (16)	153

Symmetry codes: (i) 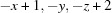; (ii) 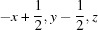; (iii) 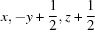.

**Fig. 1.**
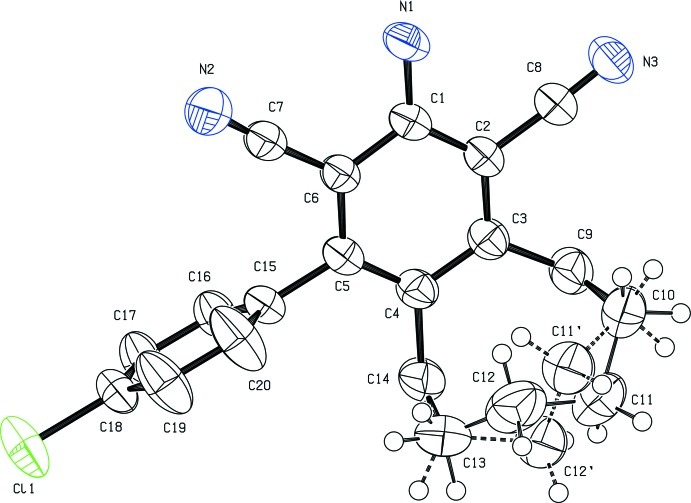
The molecular structure of the title compound, showing the atom-numbering scheme and conformational disorder. Displacement ellipsoids are drawn at the 50% probability level.

**Fig. 2.**
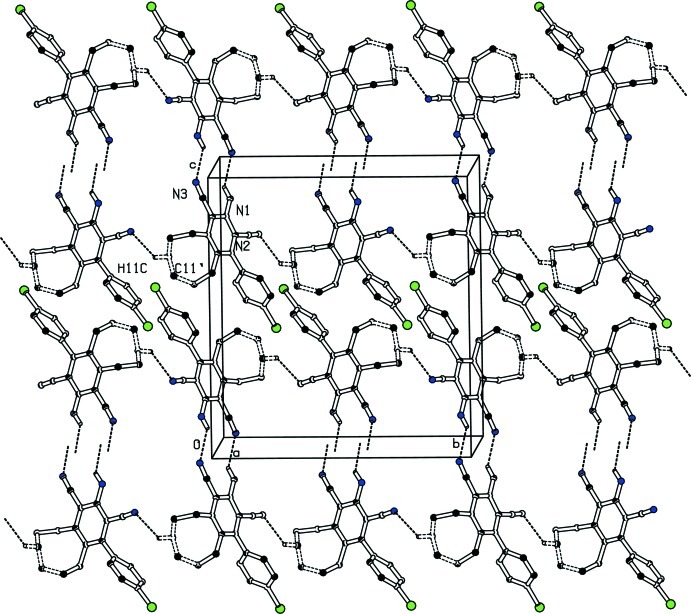
Chain linked to its inverse through N—H···N hydrogen-bonds leading to a double chain generated through characteristic R22(12) graph-set motifs across alternating centres of inversion. Non-participating H-atoms are omitted for clarity.

## supplementary crystallographic information

### Comment

Hendrickson (1967) performed energy-minimumization calculations to first establish ten types of conformers for cyclooctanes *viz*. crown (CR), chair-chair (CC), twist-chair-chair (TCC), boat (B), saddle, also known as twist-boat (TB), boat-boat (BB), boat-chair (BC), twist-boat-chair (TBC), chair (C), and twist-chair (TC). In the title compound, the torsion angle about the diene (C3\═C4) designated \t~1~ [C14—C4—C3—C9 = 3.39] shows that cyclooctene is a *cis*-conformer (Allen *et al.*, 1996).

The cyclooctene ring exhibits conformational disorder (Fig.1) that may be described as a flip-flop between twist-boat-chair (TBC) and boat-chair (BC) modes (Table 1) with the major and minor component at a ratio of about 86:14. The minor component accounts for the BC mode and seems to have induced by a C—H···N hydrogen bond which connects glide-related molecules into a chain along the *b* axis. This chain is linked to its inverse through N—H···N hydrogen-bonds lead to a double chain generated through characteristic *R*^2^_2_(12) graph-set motifs (Bernstein *et al.*, 1995) across alternating centres of inversion (Fig. 2). These double chains, characterized by the primary interactions observed among molecules in the lattice, may be regaraded as the fundamental one-dimensional building units of a two-dimensional layer which extends parallel to the *bc*-plane through N—H···N hydrogen bonds.

The Cl atoms lie almost on the b-glide plane and close to the intersections of the a- and b- glide planes. A significant non-covalent N···Cl contact of 3.261 (2) Å and a weak N—H···Cl bond is also observed (Table 2)

### Experimental

Piperidine (2ml) was added to a mixture of 3-(4-chlorophenyl)-2-cyanoacrylamide (1 mmol) , malanonitrile (1 mmol) and cyclooctanone (1 mmol) in ethanol (5ml) and heated to reflux for three hours. The reaction mixture was poured to ice. The resulting solid formed was filtered and dissolved in hot methanol. Slow evaporation of the solvent for two days resulted in crystals suitable for X-ray diffraction.

### Refinement

All the H atoms were generated geometrically and treated as riding on their respective parent atoms with default constraints using SHELXL97 (Sheldrick, 2008).

### Crystal data

**Table:**

C_20_H_18_ClN_3_	*F*(000) = 1408
*M**_r_* = 335.82	*D*_x_ = 1.249 Mg m^−^^3^
Orthorhombic, *P**b**c**a*	Mo *K*α radiation, λ = 0.71073 Å
Hall symbol: -P 2ac 2ab	Cell parameters from 3440 reflections
*a* = 11.3835 (9) Å	θ = 1.1–28.0°
*b* = 16.9840 (13) Å	µ = 0.22 mm^−^^1^
*c* = 18.4766 (14) Å	*T* = 298 K
*V* = 3572.2 (5) Å^3^	Prismatic, brown
*Z* = 8	0.28 × 0.13 × 0.10 mm

### Data collection

**Table:**

Bruker Kappa APEXII CCD diffractometer	4279 independent reflections
Radiation source: fine-focus sealed tube	3440 reflections with *I* > 2σ(*I*)
Graphite monochromator	*R*_int_ = 0.025
ω and φ scan	θ_max_ = 28.0°, θ_min_ = 2.2°
Absorption correction: multi-scan (*SADABS*; Bruker, 2009)	*h* = −14→14
*T*_min_ = 0.966, *T*_max_ = 0.978	*k* = −22→22
39132 measured reflections	*l* = −24→24

### Refinement

**Table:**

Refinement on *F*^2^	Primary atom site location: structure-invariant direct methods
Least-squares matrix: full	Secondary atom site location: difference Fourier map
*R*[*F*^2^ > 2σ(*F*^2^)] = 0.045	Hydrogen site location: inferred from neighbouring sites
*wR*(*F*^2^) = 0.137	H-atom parameters constrained
*S* = 1.04	*w* = 1/[σ^2^(*F*_o_^2^) + (0.0739*P*)^2^ + 0.6829*P*] where *P* = (*F*_o_^2^ + 2*F*_c_^2^)/3
4279 reflections	(Δ/σ)_max_ < 0.001
226 parameters	Δρ_max_ = 0.25 e Å^−^^3^
0 restraints	Δρ_min_ = −0.23 e Å^−^^3^

### Special details

**Table:**

Geometry. All e.s.d.'s (except the e.s.d. in the dihedral angle between two l.s. planes) are estimated using the full covariance matrix. The cell e.s.d.'s are taken into account individually in the estimation of e.s.d.'s in distances, angles and torsion angles; correlations between e.s.d.'s in cell parameters are only used when they are defined by crystal symmetry. An approximate (isotropic) treatment of cell e.s.d.'s is used for estimating e.s.d.'s involving l.s. planes.
Refinement. Refinement of *F*^2^ against ALL reflections. The weighted *R*-factor *wR* and goodness of fit *S* are based on *F*^2^, conventional *R*-factors *R* are based on *F*, with *F* set to zero for negative *F*^2^. The threshold expression of *F*^2^ > σ(*F*^2^) is used only for calculating *R*-factors(gt) *etc*. and is not relevant to the choice of reflections for refinement. *R*-factors based on *F*^2^ are statistically about twice as large as those based on *F*, and *R*- factors based on ALL data will be even larger.

### Fractional atomic coordinates and isotropic or equivalent isotropic displacement parameters (Å^2^)

**Table:**

	*x*	*y*	*z*	*U*_iso_*/*U*_eq_	Occ. (<1)
Cl1	0.19472 (4)	0.24645 (3)	0.44965 (3)	0.0821 (2)	
N1	0.34007 (13)	0.06894 (9)	0.89983 (6)	0.0652 (4)	
H1A	0.2861	0.1042	0.9030	0.078*	
H1B	0.3651	0.0457	0.9382	0.078*	
N2	0.17382 (14)	0.18467 (10)	0.78542 (8)	0.0701 (4)	
N3	0.54892 (13)	−0.07229 (9)	0.94323 (7)	0.0629 (4)	
C1	0.38568 (12)	0.04983 (8)	0.83396 (7)	0.0450 (3)	
C2	0.47395 (12)	−0.00739 (8)	0.82566 (7)	0.0440 (3)	
C3	0.52167 (12)	−0.02742 (8)	0.75804 (7)	0.0461 (3)	
C4	0.48105 (13)	0.01088 (8)	0.69542 (7)	0.0470 (3)	
C5	0.39315 (12)	0.06796 (8)	0.70218 (6)	0.0427 (3)	
C6	0.34465 (12)	0.08622 (8)	0.77001 (7)	0.0427 (3)	
C7	0.25038 (13)	0.14201 (9)	0.77660 (7)	0.0498 (3)	
C8	0.51593 (13)	−0.04515 (8)	0.89042 (7)	0.0492 (3)	
C9	0.61406 (14)	−0.09109 (10)	0.75523 (9)	0.0608 (4)	
H9A	0.6580	−0.0904	0.8002	0.073*	
H9B	0.6685	−0.0792	0.7164	0.073*	
C10	0.5646 (2)	−0.17414 (11)	0.74346 (11)	0.0802 (6)	
H10A	0.6238	−0.2118	0.7585	0.096*	0.859 (6)
H10B	0.4974	−0.1808	0.7751	0.096*	0.859 (6)
H10C	0.5390	−0.1952	0.7896	0.096*	0.141 (6)
H10D	0.6268	−0.2079	0.7253	0.096*	0.141 (6)
C11	0.5268 (3)	−0.19477 (15)	0.66680 (16)	0.0894 (10)	0.859 (6)
H11A	0.5101	−0.2507	0.6649	0.107*	0.859 (6)
H11B	0.5922	−0.1847	0.6345	0.107*	0.859 (6)
C12	0.4193 (3)	−0.15018 (16)	0.63835 (17)	0.0921 (10)	0.859 (6)
H12A	0.3745	−0.1307	0.6793	0.111*	0.859 (6)
H12B	0.3697	−0.1868	0.6121	0.111*	0.859 (6)
C11'	0.4529 (16)	−0.1765 (10)	0.6860 (9)	0.075 (5)*	0.141 (6)
H11C	0.4152	−0.2277	0.6865	0.091*	0.141 (6)
H11D	0.3952	−0.1366	0.6982	0.091*	0.141 (6)
C12'	0.5065 (15)	−0.1601 (8)	0.6134 (8)	0.074 (5)*	0.141 (6)
H12C	0.4884	−0.2021	0.5796	0.089*	0.141 (6)
H12D	0.5912	−0.1551	0.6173	0.089*	0.141 (6)
C13	0.4493 (3)	−0.07954 (14)	0.58781 (11)	0.0994 (8)	
H13A	0.4904	−0.0998	0.5457	0.119*	0.859 (6)
H13B	0.3762	−0.0566	0.5710	0.119*	0.859 (6)
H13C	0.4501	−0.0758	0.5354	0.119*	0.141 (6)
H13D	0.3687	−0.0758	0.6044	0.119*	0.141 (6)
C14	0.52427 (17)	−0.01371 (11)	0.62142 (9)	0.0691 (5)	
H14A	0.6049	−0.0317	0.6254	0.083*	
H14B	0.5234	0.0317	0.5895	0.083*	
C15	0.34630 (12)	0.11211 (8)	0.63809 (7)	0.0431 (3)	
C16	0.40500 (12)	0.17620 (8)	0.60985 (8)	0.0505 (3)	
H16	0.4763	0.1915	0.6300	0.061*	
C17	0.35930 (14)	0.21823 (10)	0.55193 (8)	0.0575 (4)	
H17	0.3995	0.2613	0.5332	0.069*	
C18	0.25412 (13)	0.19528 (9)	0.52285 (7)	0.0522 (3)	
C19	0.19350 (18)	0.13276 (14)	0.55030 (11)	0.0832 (7)	
H19	0.1219	0.1179	0.5303	0.100*	
C20	0.23989 (17)	0.09176 (12)	0.60831 (10)	0.0832 (7)	
H20	0.1982	0.0496	0.6276	0.100*	

### Atomic displacement parameters (Å^2^)

**Table:**

	*U*^11^	*U*^22^	*U*^33^	*U*^12^	*U*^13^	*U*^23^
Cl1	0.0710 (3)	0.1072 (4)	0.0681 (3)	0.0029 (2)	−0.0157 (2)	0.0413 (3)
N1	0.0756 (9)	0.0852 (9)	0.0350 (6)	0.0228 (7)	−0.0014 (6)	0.0009 (6)
N2	0.0683 (9)	0.0782 (10)	0.0640 (8)	0.0204 (8)	−0.0023 (7)	−0.0035 (7)
N3	0.0694 (8)	0.0719 (9)	0.0475 (7)	0.0057 (7)	−0.0065 (6)	0.0127 (6)
C1	0.0487 (7)	0.0516 (7)	0.0347 (6)	−0.0036 (6)	−0.0036 (5)	0.0009 (5)
C2	0.0457 (7)	0.0481 (7)	0.0382 (6)	−0.0039 (5)	−0.0052 (5)	0.0055 (5)
C3	0.0436 (7)	0.0508 (7)	0.0440 (7)	−0.0019 (6)	−0.0002 (5)	0.0038 (6)
C4	0.0492 (7)	0.0527 (7)	0.0391 (7)	−0.0009 (6)	0.0036 (5)	0.0040 (5)
C5	0.0455 (7)	0.0465 (7)	0.0361 (6)	−0.0072 (5)	−0.0036 (5)	0.0041 (5)
C6	0.0456 (6)	0.0451 (6)	0.0373 (6)	−0.0020 (5)	−0.0037 (5)	0.0014 (5)
C7	0.0538 (8)	0.0557 (8)	0.0399 (6)	0.0014 (6)	−0.0042 (6)	0.0004 (6)
C8	0.0503 (7)	0.0544 (8)	0.0429 (7)	−0.0025 (6)	−0.0036 (6)	0.0048 (6)
C9	0.0554 (8)	0.0723 (10)	0.0548 (8)	0.0153 (7)	0.0037 (7)	0.0072 (7)
C10	0.1036 (15)	0.0614 (10)	0.0755 (12)	0.0234 (10)	0.0093 (11)	0.0056 (9)
C11	0.109 (2)	0.0646 (13)	0.095 (2)	0.0137 (13)	0.0081 (16)	−0.0173 (13)
C12	0.097 (2)	0.0822 (16)	0.097 (2)	0.0004 (14)	−0.0155 (15)	−0.0334 (15)
C13	0.148 (2)	0.0973 (15)	0.0526 (10)	0.0276 (15)	−0.0169 (12)	−0.0201 (10)
C14	0.0842 (12)	0.0787 (11)	0.0443 (8)	0.0213 (9)	0.0168 (8)	0.0104 (7)
C15	0.0464 (7)	0.0482 (7)	0.0346 (6)	−0.0049 (5)	−0.0037 (5)	0.0034 (5)
C16	0.0462 (7)	0.0537 (7)	0.0516 (8)	−0.0076 (6)	−0.0084 (6)	0.0088 (6)
C17	0.0566 (8)	0.0567 (8)	0.0592 (9)	−0.0085 (7)	−0.0052 (7)	0.0196 (7)
C18	0.0509 (7)	0.0648 (9)	0.0409 (6)	0.0041 (6)	−0.0043 (6)	0.0129 (6)
C19	0.0713 (11)	0.1031 (14)	0.0752 (12)	−0.0364 (10)	−0.0367 (9)	0.0351 (11)
C20	0.0785 (12)	0.0942 (13)	0.0770 (12)	−0.0470 (10)	−0.0343 (10)	0.0429 (10)

### Geometric parameters (Å, º)

**Table:**

Cl1—C18	1.7441 (14)	C11—H11B	0.9700
N1—C1	1.3624 (17)	C12—C13	1.559 (4)
N1—H1A	0.8597	C12—H12A	0.9700
N1—H1B	0.8603	C12—H12B	0.9700
N2—C7	1.145 (2)	C11'—C12'	1.50 (2)
N3—C8	1.1427 (18)	C11'—H11C	0.9700
C1—C2	1.4063 (19)	C11'—H11D	0.9700
C1—C6	1.4130 (17)	C12'—C13	1.588 (15)
C2—C3	1.4042 (19)	C12'—H12C	0.9700
C2—C8	1.4391 (18)	C12'—H12D	0.9700
C3—C4	1.4056 (18)	C13—C14	1.537 (3)
C3—C9	1.509 (2)	C13—H13A	0.9700
C4—C5	1.399 (2)	C13—H13B	0.9700
C4—C14	1.512 (2)	C13—H13C	0.9700
C5—C6	1.4042 (18)	C13—H13D	0.9700
C5—C15	1.4996 (17)	C14—H14A	0.9700
C6—C7	1.437 (2)	C14—H14B	0.9700
C9—C10	1.534 (3)	C15—C20	1.375 (2)
C9—H9A	0.9700	C15—C16	1.3797 (19)
C9—H9B	0.9700	C16—C17	1.388 (2)
C10—C11	1.521 (3)	C16—H16	0.9300
C10—C11'	1.656 (17)	C17—C18	1.369 (2)
C10—H10A	0.9700	C17—H17	0.9300
C10—H10B	0.9700	C18—C19	1.364 (2)
C10—H10C	0.9700	C19—C20	1.383 (2)
C10—H10D	0.9700	C19—H19	0.9300
C11—C12	1.532 (5)	C20—H20	0.9300
C11—H11A	0.9700		
			
C1—N1—H1A	120.0	C13—C12—H12B	108.7
C1—N1—H1B	120.1	H12A—C12—H12B	107.6
H1A—N1—H1B	120.0	C12'—C11'—C10	104.9 (12)
N1—C1—C2	122.30 (12)	C12'—C11'—H11C	110.8
N1—C1—C6	121.13 (13)	C10—C11'—H11C	110.8
C2—C1—C6	116.57 (12)	C12'—C11'—H11D	110.8
C3—C2—C1	122.74 (12)	C10—C11'—H11D	110.8
C3—C2—C8	120.22 (12)	H11C—C11'—H11D	108.8
C1—C2—C8	117.03 (12)	C11'—C12'—C13	105.0 (12)
C2—C3—C4	119.54 (12)	C11'—C12'—H12C	110.7
C2—C3—C9	118.28 (12)	C13—C12'—H12C	110.7
C4—C3—C9	122.17 (13)	C11'—C12'—H12D	110.7
C5—C4—C3	118.86 (12)	C13—C12'—H12D	110.7
C5—C4—C14	120.33 (12)	H12C—C12'—H12D	108.8
C3—C4—C14	120.63 (13)	C14—C13—C12	116.08 (17)
C4—C5—C6	120.93 (11)	C14—C13—C12'	106.2 (6)
C4—C5—C15	122.04 (11)	C12—C13—C12'	41.2 (6)
C6—C5—C15	117.03 (12)	C14—C13—H13A	108.3
C5—C6—C1	121.32 (12)	C12—C13—H13A	108.3
C5—C6—C7	121.00 (11)	C12'—C13—H13A	74.7
C1—C6—C7	117.67 (12)	C14—C13—H13B	108.3
N2—C7—C6	176.28 (16)	C12—C13—H13B	108.3
N3—C8—C2	177.25 (16)	C12'—C13—H13B	142.5
C3—C9—C10	114.08 (14)	H13A—C13—H13B	107.4
C3—C9—H9A	108.7	C14—C13—H13C	110.5
C10—C9—H9A	108.7	C12—C13—H13C	130.6
C3—C9—H9B	108.7	C12'—C13—H13C	110.5
C10—C9—H9B	108.7	H13A—C13—H13C	38.7
H9A—C9—H9B	107.6	H13B—C13—H13C	70.2
C11—C10—C9	116.58 (18)	C14—C13—H13D	110.5
C11—C10—C11'	35.0 (6)	C12—C13—H13D	69.7
C9—C10—C11'	113.2 (6)	C12'—C13—H13D	110.5
C11—C10—H10A	108.1	H13A—C13—H13D	137.1
C9—C10—H10A	108.1	H13B—C13—H13D	42.6
C11'—C10—H10A	134.5	H13C—C13—H13D	108.7
C11—C10—H10B	108.1	C4—C14—C13	112.68 (16)
C9—C10—H10B	108.1	C4—C14—H14A	109.1
C11'—C10—H10B	77.2	C13—C14—H14A	109.1
H10A—C10—H10B	107.3	C4—C14—H14B	109.1
C11—C10—H10C	130.5	C13—C14—H14B	109.1
C9—C10—H10C	108.9	H14A—C14—H14B	107.8
C11'—C10—H10C	108.9	C20—C15—C16	118.28 (13)
H10A—C10—H10C	73.3	C20—C15—C5	120.25 (12)
H10B—C10—H10C	35.8	C16—C15—C5	121.39 (11)
C11—C10—H10D	75.5	C15—C16—C17	121.06 (13)
C9—C10—H10D	108.9	C15—C16—H16	119.5
C11'—C10—H10D	108.9	C17—C16—H16	119.5
H10A—C10—H10D	37.1	C18—C17—C16	118.95 (13)
H10B—C10—H10D	135.6	C18—C17—H17	120.5
H10C—C10—H10D	107.7	C16—C17—H17	120.5
C10—C11—C12	115.6 (2)	C19—C18—C17	121.21 (13)
C10—C11—H11A	108.4	C19—C18—Cl1	118.69 (12)
C12—C11—H11A	108.4	C17—C18—Cl1	120.10 (12)
C10—C11—H11B	108.4	C18—C19—C20	119.13 (15)
C12—C11—H11B	108.4	C18—C19—H19	120.4
H11A—C11—H11B	107.4	C20—C19—H19	120.4
C11—C12—C13	114.2 (3)	C15—C20—C19	121.34 (15)
C11—C12—H12A	108.7	C15—C20—H20	119.3
C13—C12—H12A	108.7	C19—C20—H20	119.3
C11—C12—H12B	108.7		
			
N1—C1—C2—C3	−179.89 (14)	C3—C9—C10—C11	77.1 (2)
C6—C1—C2—C3	1.1 (2)	C9—C10—C11—C12	−67.7 (3)
N1—C1—C2—C8	−0.6 (2)	C10—C11—C12—C13	99.9 (3)
C6—C1—C2—C8	−179.67 (12)	C11—C12—C13—C14	−60.5 (3)
C1—C2—C3—C4	0.3 (2)	C12—C13—C14—C4	−46.5 (3)
C8—C2—C3—C4	−178.99 (13)	C3—C4—C14—C13	88.71 (19)
C1—C2—C3—C9	−178.57 (13)	C3—C9—C10—C11'	38.5 (7)
C8—C2—C3—C9	2.2 (2)	C9—C10—C11'—C12'	70.7 (12)
C2—C3—C4—C5	−0.4 (2)	C10—C11'—C12'—C13	−116.6 (11)
C9—C3—C4—C5	178.44 (13)	C11'—C12'—C13—C14	85.5 (10)
C2—C3—C4—C14	−175.42 (14)	C12'—C13—C14—C4	−89.6 (6)
C3—C4—C5—C6	−0.9 (2)	C11'—C10—C11—C12	25.4 (10)
C14—C4—C5—C6	174.16 (14)	C11—C10—C11'—C12'	−33.0 (8)
C3—C4—C5—C15	179.21 (12)	C11—C12—C13—C12'	24.5 (8)
C14—C4—C5—C15	−5.7 (2)	C11'—C12'—C13—C12	−25.8 (8)
C4—C5—C6—C1	2.3 (2)	C5—C4—C14—C13	−86.27 (19)
C15—C5—C6—C1	−177.79 (11)	C4—C5—C15—C20	102.07 (19)
C4—C5—C6—C7	−176.91 (13)	C6—C5—C15—C20	−77.81 (19)
C15—C5—C6—C7	2.97 (19)	C4—C5—C15—C16	−81.27 (18)
N1—C1—C6—C5	178.61 (14)	C6—C5—C15—C16	98.85 (16)
C2—C1—C6—C5	−2.33 (19)	C20—C15—C16—C17	−1.3 (2)
N1—C1—C6—C7	−2.1 (2)	C5—C15—C16—C17	−178.01 (14)
C2—C1—C6—C7	176.94 (12)	C15—C16—C17—C18	0.1 (2)
C5—C6—C7—N2	159 (3)	C16—C17—C18—C19	0.7 (3)
C1—C6—C7—N2	−20 (3)	C16—C17—C18—Cl1	−179.58 (13)
C3—C2—C8—N3	141 (3)	C17—C18—C19—C20	−0.4 (3)
C1—C2—C8—N3	−38 (3)	Cl1—C18—C19—C20	179.96 (18)
C2—C3—C9—C10	90.97 (18)	C16—C15—C20—C19	1.7 (3)
C9—C3—C4—C14	3.4 (2)	C5—C15—C20—C19	178.4 (2)
C4—C3—C9—C10	−87.84 (18)	C18—C19—C20—C15	−0.9 (4)

### Hydrogen-bond geometry (Å, º)

**Table:**

*D*—H···*A*	*D*—H	H···*A*	*D*···*A*	*D*—H···*A*
N1—H1*B*···N3^i^	0.86	2.44	3.1636 (18)	142
C11′—H11*C*···N2^ii^	0.97	2.57	3.319 (16)	135
N1—H1*A*···Cl1^iii^	0.86	2.87	3.6628 (16)	153

Symmetry codes: (i) −*x*+1, −*y*, −*z*+2; (ii) −*x*+1/2, *y*−1/2, *z*; (iii) *x*, −*y*+1/2, *z*+1/2.
